# Sleep Apnea Symptoms and Cardiovascular Disease Risks among Haitian Medical Students

**DOI:** 10.4172/2167-0277.1000264

**Published:** 2017-04-25

**Authors:** Diana M Rosenthal, Donaldson F Conserve, Dodley Severe, Michaele A Gedeon, Ferdinand Zizi, Georges Casimir, Samy I McFarlane, Girardin Jean Louis

**Affiliations:** 1 Department of Population Health, Center for Healthful Behavior Change, NYU School of Medicine, NY, USA; 2 Department of Health Promotion, Arnold School of Public Health, Education, and Behavior, University of South Carolina, Columbia, SC, USA; 3 University of the Aristide Foundation, Haiti, USA; 4 Department of Psychiatry, SUNY Downstate Medical Center, Brooklyn, NY, USA; 5 Department of Endocrinology, SUNY Downstate Medical Center, Brooklyn, NY, USA

**Keywords:** Sleep apnea, Cardiovascular disease, Haiti, Medical students

## Abstract

Sleep apnea is a prevalent sleep disorder that disproportionately affects blacks and has been previously studied among Caribbean-born blacks in Brooklyn, New York, but there has been negligible research in the Caribbean, specifically Haiti, and developing countries on this pressing health issue. A total of 373 medical students (mean age=20.6 years ± 2.3 years) from a medical school in Haiti participated in this study. Participants were administered a questionnaire assessing their sleep health and cardiovascular outcomes. The rate of sleep apnea symptoms was: snoring (13.2%), excessive daytime sleepiness (73.7%), and difficulty maintaining sleep (25.3%). Many reported falling asleep while watching television (68.2%) or while driving (7.8%). Based on logistic regression analysis, reported nocturnal breathing pauses was the most important predictor of the likelihood of reporting a history of cardiac disease (14.96; 95% CI=1.27–76.07). Findings suggest that more aggressive effort should be made to increase screening of sleep apnea among Haitians, thereby increasing the likelihood for early detection and treatment to reduce sleep-related risk of cardiovascular disease.

## Introduction

Sleep apnea is a prevalent sleep disorder that disproportionately affects blacks [1–4]. OSA has been previously studied in Caribbean-born blacks in New York [4], but there has been negligible research in the Caribbean, specifically Haiti, and developing countries on this pressing health issue. There is significant evidence that quality of nocturnal sleep plays a role in regulating Blood Pressure (BP) levels based on evidence that shows a significant association between poor nocturnal sleep and non-dipping behavior as well as the presence of sleep apnea [5]. Such evidence has concluded that OSA is an indicator of cardio metabolic risk.

In Haiti, there are a number of structural and historical factors that may play role in producing sleep apnea. For instance, Haiti is the poorest country in the Americas and one of the poorest in the world (with a GDP per capita of US $846 in 2014) with significant needs in basic services. According to the World Health Organization (WHO), the total expenditure on health per capita (Intl $, 2014) is 131 and the total expenditure on health as percent of GDP is 7.6% in 2014 [6]. In addition, Haiti was struck by a 7.0 magnitude earthquake that left much devastation and long term psychological effects on the survivors. Although Haiti has been recovering from the earthquake, a number of challenges remain and survivors may still be suffering from posttraumatic stress disorder (PTSD). In a study conducted among 246 Haitian students 2 years after the earthquake, a prevalence of 36% for PTSD and 32% for depression was reported [7] and another study found higher PTSD symptoms among female university students than male [8]. Findings from a recent systematic review suggest that sleep apnea is positively associated with PTSD and major depressive disorder [9]. In 2015, five years after the earthquake, the reported life expectancy at birth in Haiti was 62/66 (m/f) [10]. According to WHO, 48% of total deaths in Haiti are attributed to non-communicable diseases (NCDs); furthermore, the probability of dying from one of the four main NCDs (cardiovascular, respiratory, diabetes, and cancer) is 24% for Haitians between ages 30 and 70 [6].

The probability of death from one of the four main NCDs for Haitians may be influenced by the structural and historical factors associated with the presence of sleep fragmentation and sleep apnea in various communities. Research conducted in a Haitian community without electricity showed that sleep fragmentation differed by age groups; however, this Haitian sample reported a greater average time in bed than reported for developed countries like the United States (9.3 v*s.* 7 hours to 8 hours), but the average sleep duration of this sample was shorter at 7 hours [11]. Further research is needed to determine other co-contributing factors to the presence of sleep apnea including socio-demographic and cultural determinants, and if sleep apnea is associated with the high NCD mortality rate in Haiti. The aim of this study is to examine the presence of sleep fragmentation and sleep apnea in a sample of Haitian medical students, and observe the factors potentially impacting the occurrence of this health phenomenon.

## Methods

A total of 373 medical students participated in the study. The average age was 20.6 years ± 2.3 years (range=16 years to 35 years). Of the sample, 67.4% were women and 32.6% were men. The data was collected in two waves among students at the Medical School in Port-au-Prince, Haiti. Eligible participants were approached by a health educator (or medical student) who explained the purpose of the study and provided answers to questions raised by the participants. The health educator assisted all participants in completing the questionnaires. Questionnaires were available in both French and Haitian Creole, requiring approximately 15 minutes to complete.

### Study variables

Self-reported data relating to history of hypertension and cardiac disease were collected in the questionnaire. For this study, we asked participants several questions such as: “Do you have a history of cardiac problems?” with a binary (yes/no) response option. Participants were also asked to give yes/no answers based on whether they experienced habitual snoring, excessive daytime sleepiness, and sleep fragmentation. Subjective questions as well as self-rated items on health status, sleep apnea symptoms, insomnia, and daytime functioning were also asked. For example, participants were asked to rate their health status from “excellent” to “poor” and rate their satisfaction with their current sleep status from “very satisfactory” to “very trouble.” The questionnaire also assessed the number of hours spent in bed, number of hours actually spent sleeping, and daytime napping.

### Statistical analysis

The present analysis examined sleep characteristics and assessed their associations with cardiovascular outcomes. Frequency and measures of central tendency were used to describe the sample. In preliminary analyses, Pearson and Spearman correlations were used to explore relationships between variables of interest. Relationships of sleep characteristics with cardiovascular outcomes (i.e., hypertension and heart disease) were examined with multiple logistic regression analysis. Factors were selected based on their theoretical importance. Demographic and health risks were adjusted in the regression model. SPSS 21.0 was used for the statistical analyses.

## Results

The rate of OSA symptoms in this sample was: snoring (13.2%), witnessed apneas (25.7%), and excessive daytime sleepiness (73.7%). Many of the respondents indicated falling asleep while watching television (68.2%) or while driving (7.8%). Overall, 27.8% reported difficulty initiating sleep, 25.3% difficulty maintaining sleep, and 56.6% early morning awakening; 45.5% reported excessive tossing and turning while sleeping; 61.3% indicated daytime napping and 9.8% used sleep medicine. Of the sample, the average time spent in bed at night was 6.48 hours ± 1.9 hours and the average time spent in bed sleeping at night was 5.99 hours ± 1.5 hours. For the cardiovascular outcomes, 25.5% reported a history of hypertension and 10.5% a history of heart disease. More than half (61.5%) reported difficulty concentrating during the day and 68.5% rated their health status as good to excellent and 31.5% as fair to poor. Most of the participants (62.1%) were satisfied with their habitual sleep. The average weight and height of the participants were 69.5 kg ± 27.5 kg, 1.63 m ± 0.17 m, respectively.

In Table 1, we contrast sleep characteristics of participants reporting adverse cardiovascular outcomes (i.e., hypertension or heart disease). Based on logistic regression analysis, difficulty breathing at night was the most important predictor of the likelihood of reporting a history of cardiac problems. The corresponding multivariate-adjusted odds ratio was 14.96 (95% CI=1.27–76.07). Sleep factors were not significantly associated with the presence of hypertension (Table 1).

## Discussion

To our knowledge, this is the first study to examine symptoms of OSA and self-reported cardiovascular outcomes among Caribbean men and women residing in Haiti. The main finding of the study is that a significant number of Haitians reported sleep apnea symptoms (i.e., snoring and excessive daytime sleepiness) and that difficulty breathing at night is a strong predictor of the likelihood of reporting a history of heart disease. Excessive daytime sleepiness, another symptom highly suggestive of greater sleep apnea risk, was also more common among medical students in Haiti. Specifically, 73.7% of the participants reported excessive daytime sleepiness. By contrast, the estimated rate of daytime sleepiness in the 2005 Sleep in America poll was 27% [12]. Comparatively, estimates of daytime sleepiness in Sweden, France, and in the UK were 16%, 20%, and 15%, respectively [13–16]. Furthermore, the rates of daytime sleepiness in our study were similar to the rates among Caribbean-born blacks in Brooklyn, NY (33%). However, these rates are higher than those generally observed for blacks (19%) in the U.S. [17], who typically experience more severe daytime sleepiness than do age-matched whites [18,19]. These findings support the notion that blacks do not constitute a homogenous group regarding snoring and daytime sleepiness, two of the most frequent symptoms of sleep apnea.

Students in our sample reported an average time spent in bed at night of 6.48 hours ± 1.9 hours, and the average time spent in bed sleeping at night was 5.89 hours ± 1.5 hours. This compares to an average sleep time of 9.3 hours ± 1.2 hours and 7.0 hours ± 1.0 hours, respectively with a previous study [11]. This noticeable discrepancy in bed and sleep time is possibly due to the difference in the study samples. It can be expected that medical students sleep less and spend more time studying, while community members in the other may have a more sedentary lifestyle without electricity [11]. In addition, our sample consisted of a younger age group, while the participants in the other study ranged from 18 years to ≥ 65 years of age. In contrast, in a Jamaican lifestyle survey conducted with 2,432 participants, they reported sleeping 8.2 hours ± 1.8 hours [20].

Similar to other studies, we found an association between history of cardiac disease and sleep apnea symptoms, although the confidence interval for the regression model suggests a great deal of variability [21–23]. Furthermore, the Sleep Heart Health Study showed that sleep apnea increases the risk of heart failure by 140%, the risk of stroke by 60%, and the risk of coronary heart disease by 30% [24]. One implication of these findings is that public health efforts should promote adequate screening and timely diagnosis of sleep apnea among Haitians in order to prevent heart disease. Caribbean patients with a history of heart disease should receive a brief screening for sleep apnea while attending regular visits in primary-care facilities. Screening instruments should be made available and can be easily administered by the medical staff. Appropriate referrals for comprehensive sleep assessment should be encouraged.

## Conclusion

As expected, sleep apnea risk factors, specifically difficulty breathing at night, was a predictor of heart disease. In addition, the Haitian participants reported high rates of sleep apnea symptoms (i.e., snoring, difficulty breathing at night and excessive daytime sleepiness) similar to Caribbean-born blacks studied in the U.S. Of note, since Haitian medical students in our sample represent a particular age group and occupational setting, estimates from our sample are not generalizable to population-based estimates.

Based on the geographic location, almost all of the medical students were from Port-au-Prince area, so it is not a reflection of the entire country. Likewise, our sample comprised young medical students who were not seeking sleep services. Our data suggests that blacks in developing countries may be at risk of developing sleep apnea. Furthermore, based on these findings, it can be said that Caribbean individuals at-risk for OSA can experience increased risks when they immigrate to the U.S. Our previous study showed that Caribbean-born blacks were at greater risk of developing sleep apnea compared with US-born blacks [4]. This suggests that more aggressive effort should be made to increase screening rates for sleep apnea in the Caribbean, thereby increasing the likelihood for early detection and treatment to prevent cardiovascular complications. Future studies should assess sleep patterns among Haitians using more robust epidemiologic methodologies.

## Figures and Tables

**Table 1:** Sleep characteristics of participants reporting adverse cardiovascular outcomes.

Variable	N	No (%)	Yes (%)
Snoring	365	317 (86.85)	48 (13.15)
Witnessed Apneas	366	272 (74.3)	94 (25.7)
Excessive Daytime Sleepiness	361	95 (26.3)	266 (73.7)
Falling Asleep Watching TV	368	117 (31.8)	251 (68.2)
Falling Asleep Driving	256	236 (92.2)	20 (7.8)
Difficulty Initiating Sleep	367	265 (72.2)	102 (27.8)
Difficulty Maintaining Sleep	363	271 (74.7)	92 (25.3)
Early Morning Awakening	357	155 (43.4)	202 (56.6)
Excessive Tossing and Turning	365	199 (54.5)	166 (45.5)
Daytime Napping	362	140 (38.7)	222 (61.3)
Use of Sleep Medicine	368	332 (90.2)	36 (9.8)

Adverse cardiovascular outcomes were reported as binary (yes/no) responses. The number and percentage of these self-reported outcomes in our sample of medical students are presented respectively in Table 1.

## References

[1] Smedley BD, Stith AY, Nelson AR (2002). Unequal treatment: Confronting racial and ethnic disparities in health care.

[2] Williams DR (2003). The health of men: structured inequalities and opportunities. Am J Public Health.

[3] Williams NJ, Jean Louis G, Ravenell J, Seixas A, Islam N (2016). A community-oriented framework to increase screening and treatment of obstructive sleep apnea among blacks. Sleep Med.

[4] Zizi F, Jean Louis G, Fernandez S, von Gizycki H, Lazar JM (2008). Symptoms of obstructive sleep apnea in a Caribbean sample. Sleep Breath.

[5] Silva A, Moreira C, Bicho M, Paiva T, Clara J (2000). Nocturnal sleep quality and circadian blood pressure variation. Rev Port Cardiol.

[6] WHO (2014). Noncommunicable diseases (NCD) country profiles. Haiti.

[7] Silvestre G, Anacréon P, Théodore M, Silvestre E, Garcia Dubus E (2014). Risk Factors for Posttraumatic Stress Disorder in Haitian Students. Psychology.

[8] Burnett HJ, Helm H (2013). Relationship between posttraumatic stress disorder, resilience, and religious orientation and practices among university student earthquake survivors in Haiti. Int J Emerg Ment Health.

[9] Gupta MA, Simpson FC (2015). Obstructive sleep apnea and psychiatric disorders: a systematic review. J Clin Sleep Med.

[10] World Health Organization (WHO) (2015). Haiti: Statistics.

[11] Knutson KL (2014). Sleep duration, quality, and timing and their associations with age in a community without electricity in Haiti. Am J Hum Bio.

[12] Friedman M, Bliznikas D, Klein M, Duggal P, Somenek M (2006). Comparison of the incidences of obstructive sleep apnea-hypopnea syndrome in African-Americans versus Caucasian-Americans. Otolaryngol Head Neck Surg.

[13] Ohayon MM, Caulet M, Philip P, Guilleminault C, Priest RG (1997). How sleep and mental disorders are related to complaints of daytime sleepiness. Arch Intern Med.

[14] Ohayon MM, Guilleminault C, Priest RG, Caulet M (1997). Snoring and breathing pauses during sleep: telephone interview survey of a United Kingdom population sample. BMJ.

[15] Cumberbatch CG, Younger NO, Ferguson TS, McFarlane SR, Francis DK (2011). Reported hours of sleep, diabetes prevalence and glucose control in jamaican adults: analysis from the Jamaica lifestyle survey 2007–2008. Int J Endocrinol.

[16] Soldatos CR, Allaert FA, Ohta T, Dikeos DG (2005). How do individuals sleep around the world? Results from a single-day survey in ten countries. Sleep Med.

[17] Qureshi AI, Giles WH, Croft JB, Bliwise DL (1997). Habitual sleep patterns and risk for stroke and coronary heart disease A 10-year follow-up from NHANES I. Neurology.

[18] Ancoli Israel S, Klauber MR, Stepnowsky C, Estline E, Chinn A (1995). Sleep-disordered breathing in African-American elderly. Am J Respir Crit Care Med.

[19] Durrence HH, Lichstein KL (2006). The sleep of African Americans: a comparative review. Behav Sleep Med.

[20] Findley LJ, Weiss JW, Jabour ER (1991). Drivers with untreated sleep apnea: a cause of death and serious injury. Arch Intern Med.

[21] USDHHS (1993). Wake Up America National Sleep Alert.

[22] Kiely J, McNicholas W (2000). Cardiovascular risk factors in patients with obstructive sleep apnoea syndrome. Eur Respir J.

[23] Mehra R, Benjamin EJ, Shahar E, Gottlieb DJ, Nawabit R (2006). Association of nocturnal arrhythmias with sleep-disordered breathing: The Sleep Heart Health Study. Am J Respir Crit Care Med.

[24] Wright J, Johns R, Watt I, Melville A, Sheldon T (1997). Health effects of obstructive sleep apnoea and the effectiveness of continuous positive airways pressure: a systematic review of the research evidence. BMJ.

